# A Comprehensive Study of One-Step Selenization Process for Cu(In_1−*x*_Ga_*x*_)Se_2_ Thin Film Solar Cells

**DOI:** 10.1186/s11671-017-1993-0

**Published:** 2017-03-21

**Authors:** Shih-Chen Chen, Sheng-Wen Wang, Shou-Yi Kuo, Jenh-Yih Juang, Po-Tsung Lee, Chih Wei Luo, Kaung-Hsiung Wu, Hao-Chung Kuo

**Affiliations:** 1 Department of Electrophysics, National Chiao-Tung University, Hsinchu, Taiwan; 2 Department of Photonics and Institute of Electro-Optical Engineering, National Chiao-Tung University, Hsinchu, Taiwan; 3 Department of Electronic Engineering, Chang Gung University, Taoyuan, Taiwan; 4 Department of Nuclear Medicine, Chang Gung Memorial Hospital, Taoyuan, Taiwan

**Keywords:** Selenization, CIGS, Solar cell, MoSe_2_, Pump-probe spectroscopy

## Abstract

In this work, aiming at developing a rapid and environmental-friendly process for fabricating CuIn_1−*x*_Ga_*x*_Se_2_ (CIGS) solar cells, we demonstrated the one-step selenization process by using selenium vapor as the atmospheric gas instead of the commonly used H_2_Se gas. The photoluminescence (PL) characteristics indicate that there exists an optimal location with superior crystalline quality in the CIGS thin films obtained by one-step selenization. The energy dispersive spectroscopy (EDS) reveals that the Ga lateral distribution in the one-step selenized CIGS thin film is intimately correlated to the blue-shifted PL spectra. The surface morphologies examined by scanning electron microscope (SEM) further suggested that voids and binary phase commonly existing in CIGS films could be successfully eliminated by the present one-step selenization process. The agglomeration phenomenon attributable to the formation of MoSe_2_ layer was also observed. Due to the significant microstructural improvement, the current–voltage (*J*-*V*) characteristics and external quantum efficiency (EQE) of the devices made of the present CIGS films have exhibited the remarkable carrier transportation characteristics and photon utilization at the optimal location, resulting in a high conversion efficiency of 11.28%. Correlations between the defect states and device performance of the one-step selenized CIGS thin film were convincingly delineated by femtosecond pump-probe spectroscopy.

## Background

CuIn_1−*x*_Ga_*x*_Se_2_ (CIGS) has emerged as one of the most promising materials for low cost and high-efficiency thin-film solar cells. CIGS possesses superior optical properties such as high absorption coefficient (*α* ~ 10^5^ cm^−1^), long-term stability, and high radiation tolerance, due to that it belongs to the family of direct band gap chalcopyrite semiconductor compounds. Moreover, its band gap can be engineered by the partial substitution of indium by gallium, which further renders the flexibility of manipulating the optical absorption [1]. Nowadays, various methods for preparing CIGS absorber layers have been implemented, including vacuum processes (co-evaporation [2], sputtering [3], and pulsed laser deposition [4]) and nonvacuum processes (ink-printing [5] and electrochemical deposition [6]). However, the high quality CIGS thin films for laboratory-scale or module-level solar cells are usually prepared by co-evaporation or sputtering processes. In general, co-evaporation and sputtering processes for growing CIGS thin films both include the metallic precursor deposition of Cu–In–Ga alloy and the subsequent two-step selenization process, which consists of an initial low temperature selenization in H_2_Se atmosphere followed by a high temperature annealing in inert gas. By precisely controlling the parameters of selenization, it has been demonstrated that homogeneous CIGS films with tunable in-depth Ga distribution can be obtained, such that the open-circuit voltage (*V*_oc_) of the devices can be increased due to the formation of the Ga-rich surface layer [7, 8].

However, owing to the increasing environmental concerns on the green energy issues, the researchers are still seeking for yet more environmental friendly and simpler selenization processes for preparing CIGS thin films. To this respect, here, we report a one-step selenization process by using selenium (Se) vapor as the atmospheric gas instead of the commonly used H_2_Se gas. The photoluminescence (PL) intensity mapping image and spectra of the one-step selenized CIGS thin film indicate that there exists an optimal location exhibiting completed stoichiometric reaction with the introduced Se vapor flux. The lateral distribution of Ga and the inhomogeneous surface morphology of the one-step selenized CIGS film were examined by energy dispersive spectroscopy (EDS) and scanning electron microscope (SEM) to delineate the prominent role played by Ga-doping. The current–voltage (*J*-*V*) characteristics and external quantum efficiency (EQE) of the devices made from different locations of the one-step selenized CIGS thin film were measured to reveal its correlations with film stoichiometry. Finally, the ultrafast carrier dynamics at different locations of the CIGS film were probed with femtosecond (fs) optical-pump optical-probe spectroscopy to elaborate the key mechanism governing the efficiency of CIGS solar cells.

## Methods

### Farbrication of CIGS Thin Film Solar Cells

**Fig. 1:**
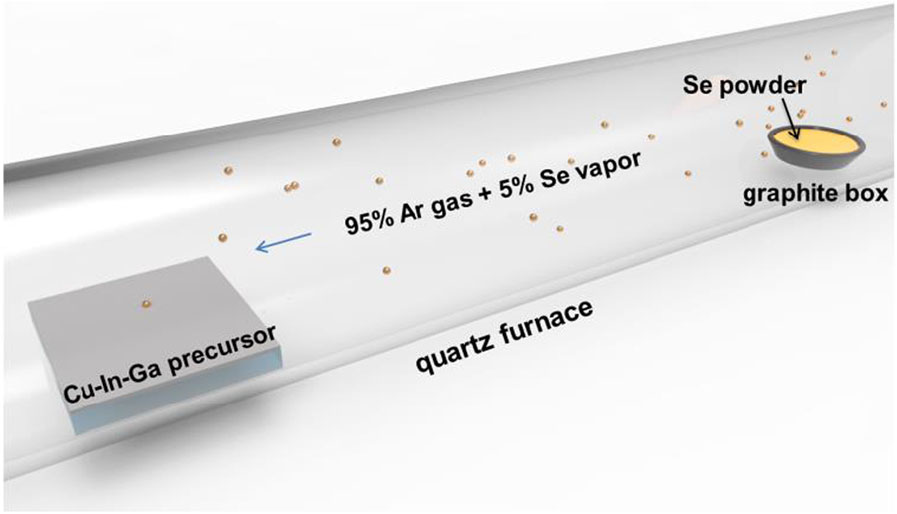
Schematic drawing of the one-step selenization process

The metallic precursors, Cu_0.75_Ga_0.25_ and elemental In targets, were deposited on a Mo-coated soda-lime glass (SLG) substrate (100 mm × 100 mm) by dc-magnetron sputtering. The precursor elements were co-sputtered at room temperature, and the substrate was rotated continuously during deposition. The Cu–In–Ga alloy (thickness ~0.6 μm) was formed at the working pressure of 3 × 10^−3^ mbar. The Cu/(In + Ga) and Ga/(Ga + In) atomic ratios of the precursor were 0.9 and 0.25, respectively. Subsequently, the Cu–In–Ga metallic precursor was inserted into a quartz furnace for one-step selenization process (see the schematic illustration shown Fig. 1). The reaction under the Se vapor was conducted in a horizontal tube reactor at pressure of 450–500 Torr. The Se vapor was diluted in the Ar gas atmosphere with 5% molar concentration during the one-step selenization process. The substrate temperature was raised from room temperature to 550 °C within 25 min. The selenization reaction time was longer than 20 min at 550 °C. For device fabrications, a CdS buffer layer (50–70 nm) was deposited by chemical bath deposition (CBD) for *p-n* junction formation. An insulating ZnO (i-ZnO) winder layer and an Al-doped ZnO (AZO) transparent conductive oxide layer were capped on the *p-n* junction. Finally, the silver grid pattern was coated as the top layer by the printing process. The different CIGS solar cells were obtained from the 100 × 100 mm^2^ substrate using a mechanical scribe.

### Characterizations

A 635-nm diode laser was employed to excite the sample for PL measurements. The PL intensity mapping image and spectra were acquired by infrared (IR) camera and photomultiplier tube (PMT), respectively. The surface morphologies of the CIGS thin film were examined by scanning electron microscopy (SEM, JSE-7001, JEOL, Tokyo, Japan). Composition information of the samples were analyzed by using energy dispersive spectrometer (EDS, INCA analysis system, Oxford Instruments, Oxford shire, UK) at an accelerating voltage of 15 kV with the sampling depth of about 1 μm. *J*-*V* characteristics of the devices were measured following the procedures described in the international standard CEI IEC 60904-1. The cells were characterized under a simulated Air Mass 1.5, Global (AM1.5G) illumination with a power of 1000 W/m^2^. The external quantum efficiency (EQE) of devices was measured by a 300-W xenon lamp (Newport 66984) light source with a monochromator (Newprot 74112). The EQE data were acquired using a lock-in amplifier (Stanford Research System, SR830) with an optical chopper unit (SR540) operated at 260 Hz chopping frequency and a 1Ω resistor in shunt connection to convert the photocurrent into voltage.

## Results and Discussion

**Fig. 2:**
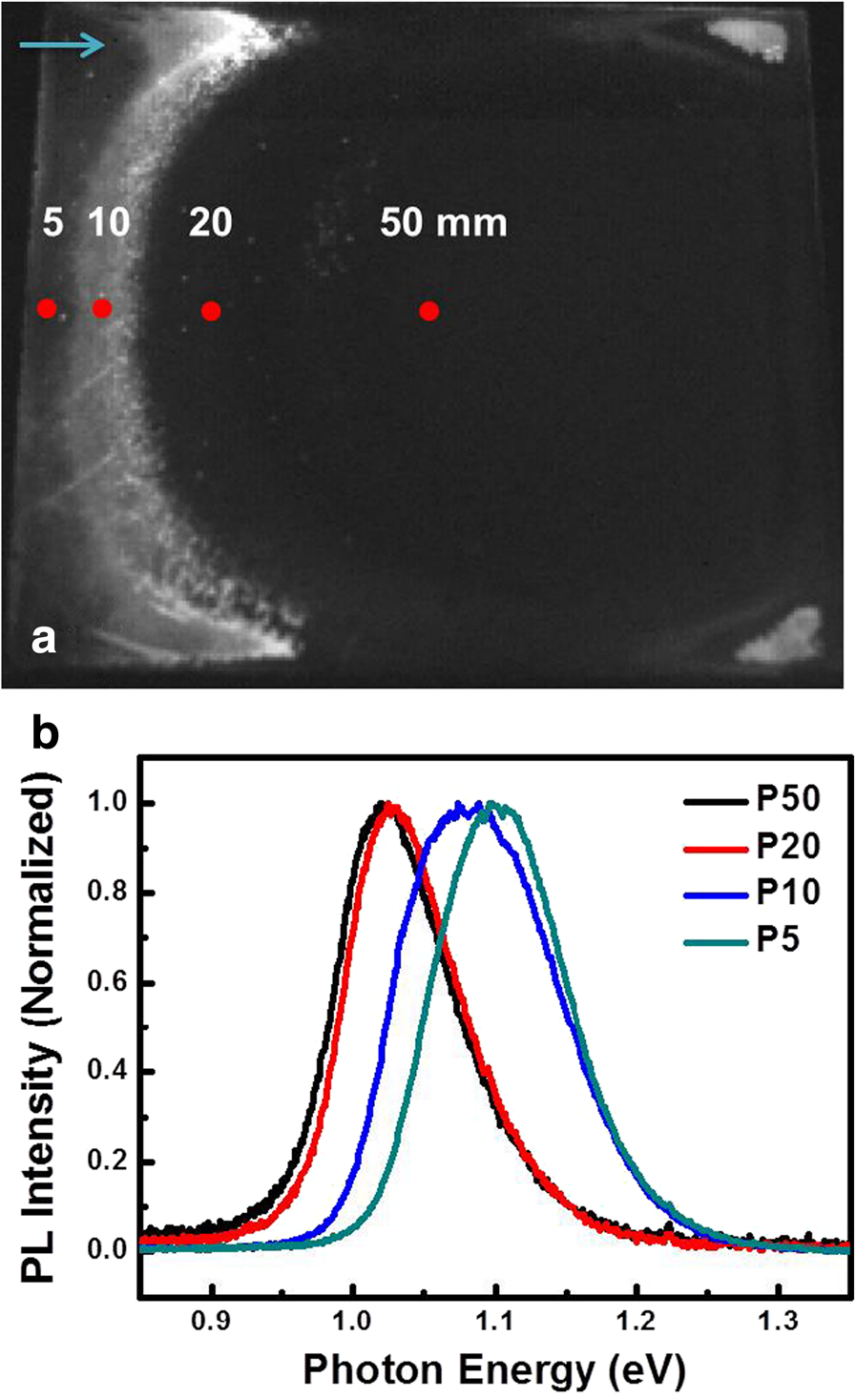
The PL **a** intensity mapping image and **b** spectra of one-step selenized CIGS thin film

Figure 2a shows the PL intensity mapping image over an area of 100 × 100 mm^2^ CIGS thin film prepared by the one-step selenization process measured at room temperature with an excitation power of 3 mW. It can be seen that the one-step selenization process has resulted in a rather non-uniform distribution of PL intensity. Figure 2b displays the room-temperature PL spectrum of each point taken at different distances (5, 10, 20, 50 mm) from the left edge (The incoming direction of Se vapor flux is denoted by the arrow.) of the sample (indicated by the red dots shown in Fig. 2a) and are denoted as P5, P10, P20, and P50, respectively. The energy of the PL emission peak was within the range of about 1.03 ~ 1.12 eV, agreeing well with the band gap of CIGS (~1.1 eV) [9]. It is noted that the noticeable blue shift of PL emission peak energy from P50 to P5 implicates the variation of compositions at these locations. The elemental compositions at different film locations determined by EDS analyses are listed in Table 1. From the results, it is evident that at P20 and P50 the film is essentially gallium-free (Ga = 0), presumably due to the off-stoichiometry reaction resulted from unavailable in-depth Ga distribution. Nevertheless, the Ga/(Ga + In) ratio is substantially increased to 0.16 and 0.18 for P10 and P5, respectively. Such a stoichiometry change may account for the blue-shifted PL spectra displayed in Fig. 2b. In contrast, the amount of In exhibits a changing trend opposite to that of Ga. This phenomenon of inhomogeneous selenization can be attributed to the cracked flux of Se vapor during the reaction [10].

**Table 1:** Compositions for different locations at one-step selenized CIGS thin film

Location (mm)	Cu (at.%)	Se (at.%)	Ga (at.%)	In (at.%)	$\frac{\mathrm{Cu}}{\left(\mathrm{Ga}+\mathrm{In}\right)}$	$\frac{\mathrm{Ga}}{\left(\mathrm{Ga}+\mathrm{In}\right)}$
5	21.39	53.50	4.54	20.57	0.85	0.18
10	21.31	54.17	3.87	20.65	0.87	0.16
20	24.03	49.56	0	26.41	0.91	0
50	25.46	48.53	0	26.01	0.98	0

**Fig. 3:**
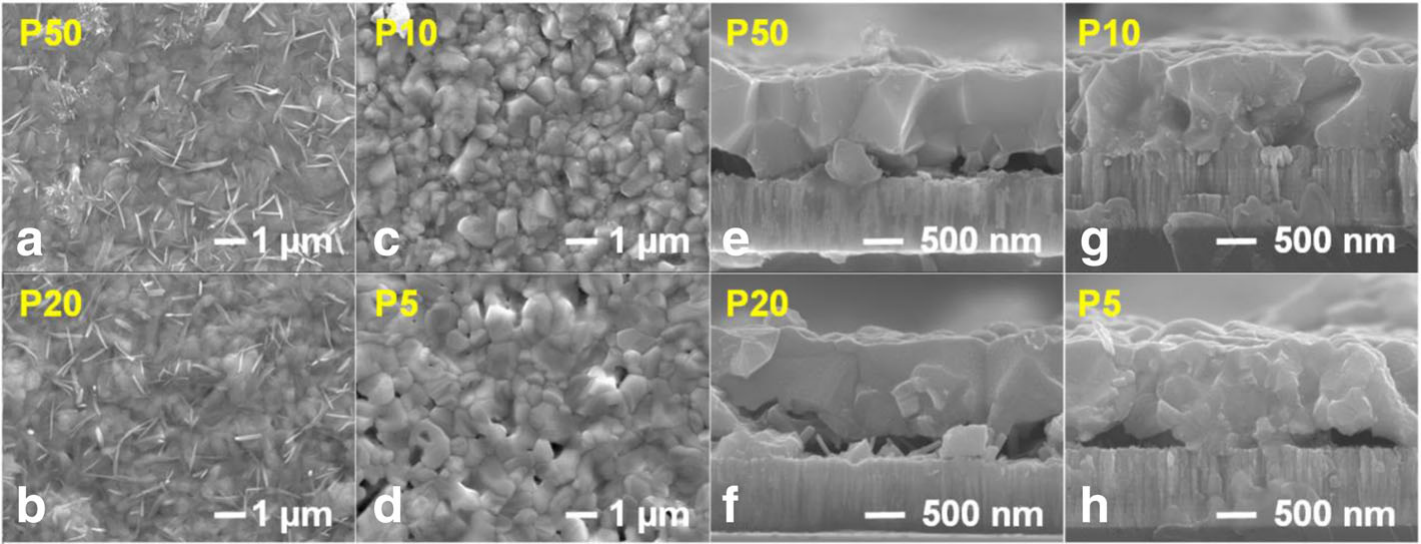
The *top-view* (**a**–**d**) and *side-view* (**e**–**h**) SEM images for different locations of one-step selenized CIGS

Figure 3 shows the top-view and side-view SEM images taken at different locations of the CIGS thin films prepared by the one-step selenization process. Figure 3a–d shows the top-view SEM images for P50, P20, P10, and P5, respectively. From the surface morphology, it is apparent that at P50 (Fig. 2a) and P20 (Fig. 2b) the grain structure consists of irregular-shape grains and some distributed needle-like grains, suggesting that in these regions the film is mainly a mixture of off-stoichiometry binary phases (i.e., InSe and In_2_Se) [11, 12]. The grains of irregular morphology at P50 and P20 may also relate to the residual Cu_2_Se binary phase [13]. In any case, the appearance of those binary phases is also consistent with the gallium-free phenomenon observed at P50 and P20. Moreover, the voids between CIGS thin film and Mo back contact were observed at P50 and P20 (see Fig. 3e, f), which can further lead to deteriorated carrier transportation, and hence the performance of the devices. In contrast, the chunk-shape grain morphology observed at P10 and P5 (see Fig. 3c, d) suggests the Cu-poor characteristic in these regions [7], which is also consistent with the lower Cu/(In + Ga) ratios, 0.87 and 0.85, for P10 and P5, respectively. The cross-sectional view SEM image of P10 (Fig. 3g) exhibits a feature of grain agglomeration, which can be attributed to the formation of the MoSe_2_ layer between CIGS thin film and Mo back contact [14, 15]. It is believed that, by introducing Se vapor flux with the present scheme, complete selenization was achieved at an optimal location near P10, which, in turn, results in the superior crystalline quality as well as the higher PL intensity observed in Fig. 2a.

**Fig. 4:**
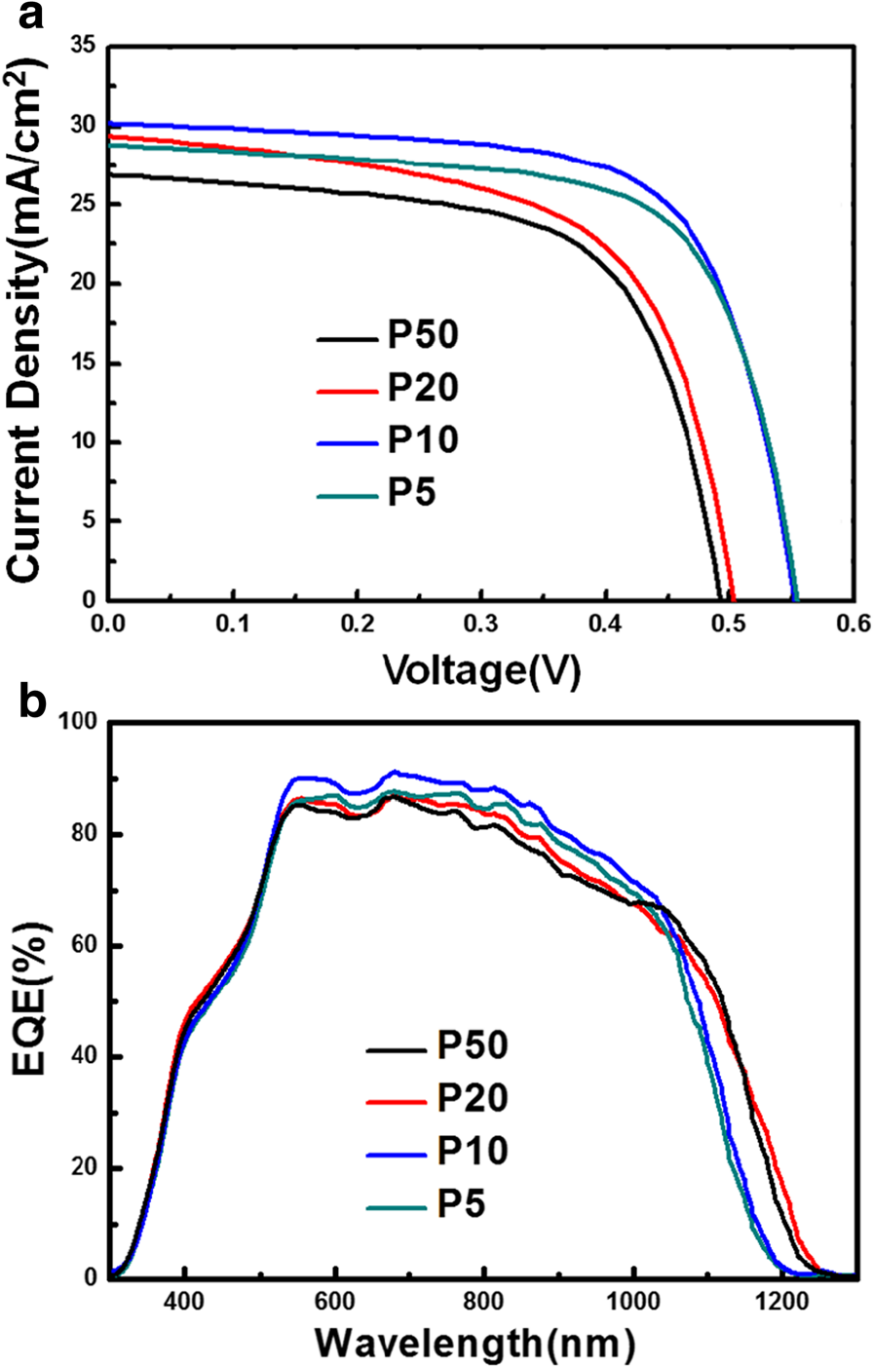
**a** *J-V* and **b** EQE measurements for different locations of one-step selenized CIGS

Figure 4a exhibits the current–voltage (*J-V*) characteristics for the devices made from areas at different locations of the one-step selenized CIGS thin film. The corresponding device performance parameters are listed in Table 2. The larger *V*_oc_’s obtained for devices made of films at P10 and P5 can be related to the enlarged bandgap resulted from increasing Ga content [16]. In particular, it is noted that the fill factor (FF) is significantly enhanced at location of P10, presumably owing to the agglomeration of grain structures and the formation of the MoSe_2_ layer, which may have greatly modified the carrier transportation. Consequently, the highest conversion efficiency of 11.28% has been achieved at location of P10. (The conversion efficiencies of two-step selenized CIGS thin film solar cells with the same Cu–In–Ga precursor were averagely distributed at 11%.) The external quantum efficiency (EQE) measurements were also conducted at the four positions as shown in Fig. 4b. The EQE loss observed in the wavelength region of 400–500 nm is due to the absorption of CdS buffer layer. At long wavelength region, the cut-off wavelengths of P5 and P10 were blue-shifted that are consistent with the enlarged band gap of CIGS thin films (Fig. 2b). It is also evident that the modification of grain structures and stoichiometric composition at P10 are beneficial to a broadband enhancement of photon utilization, leading to the higher short-circuit current density exceeding 30 mA/cm^2^.

**Table 2:** *J-V* characteristics for different locations of one-step selenized CIGS

Location	*V*_oc_(mV)	*J*_sc_(mA/cm^2^)	FF(%)	Efficiency(%)
P5	556	28.76	67	10.75
P10	553	30.18	68	11.28
P20	505	29.39	60	8.95
P50	494	26.91	64	8.48

**Fig. 5:**
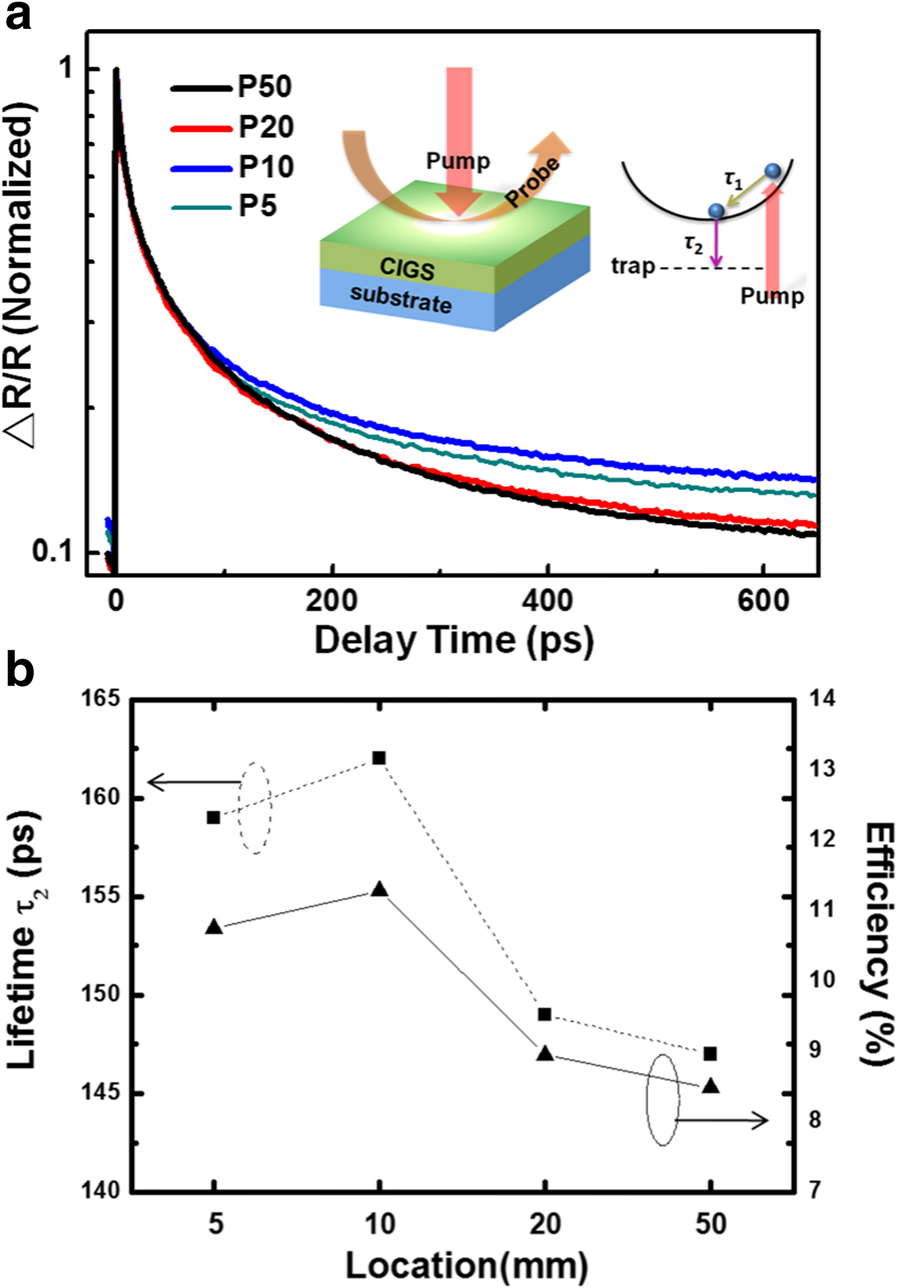
**a** The reflectivity transient (△R/R) and **b** the correlation between defect-related carrier lifetime and conversion efficiency

In order to investigate the defects existing in the one-step selenized CIGS thin film, the femtosecond pump-probe spectroscopy measurement was carried out, which is a powerful technique to delineate non-equilibrium carrier dynamics in the present CIGS films [17, 18]. Upon the illumination of the pumping pulses from the femtosecond laser, a large amount of carriers are expected to be excited in the CIGS thin film. The subsequent carrier relaxation processes are then to be probed by a sequence of probing pulses, which are simultaneously reflected on the reflectivity transient (△R/R). The semi-log plots of the ΔR/R for different locations obtained at the one-step selenized CIGS are plotted in Fig. 5a. The curves are fitted with a bi-exponential decay function, [A_1_exp(−t/*τ*_1_) + A_2_exp(−t/*τ*_2_)], with *τ*_1_ and *τ*_2_ being related to hot carrier relaxation and defect-related recombination, respectively. It is apparent from the results that the longest defect-related carrier lifetime (*τ*_2_) is obtained at location of P10, suggesting the minimum density of defect states in this region [17], which is well consistent with the much superior crystalline quality in this region described above. The magnitudes of the defect-related carrier lifetime (*τ*_2_) are further compared with conversion efficiency in Fig. 5b. The nearly one-to-one correspondence has convincingly confirmed the intimate correlation between the density of defect states and the conversion efficiency of the devices.

## Conclusions

In summary, we have successfully demonstrated that the one-step selenization process using selenium vapor instead of H_2_Se can meet the demand of high efficiency and environmental friendly method for preparing high-quality CIGS thin films. The photoluminescence intensity mapping image obtained from the one-step selenized CIGS thin film suggested that there exists an optimal location (P10) for obtaining CIGS film with superior crystalline quality. The blue-shifted photoluminescence spectra obtained from the regions at P5 and P10 are consistent with the lateral distribution of Ga element as revealed by energy dispersive spectroscopy (EDS). According to the surface morphologies examined by scanning electron microscope, it is evident that the binary phase and voids of one-step selenized CIGS film can be eliminated at the P10, with agglomeration of grain and the formation of a MoSe_2_ layer. Consequently, the current-voltage characteristics and external quantum efficiency of the devices made of film regions near P10 exhibited characteristics of highly efficient carrier transportation and remarkable photon utilization, which are intimately related to the improvements of grain structures and stoichiometric composition. Finally, the femtosecond pump-probe spectroscopy studies unambiguously revealed the close correlations between the defect-related carrier lifetime, density of defect states, and the conversion efficiency of the devices. The present results indicate that the one-step selenization process might serve as a viable avenue for developing the high-efficiency CIGS solar cells.

## Abbreviations

CBD: Chemical bath deposition
CIGS: CuIn_1−*x*_Ga_*x*_Se_2_
EDS: Energy dispersive spectrum
EQE: External quantum efficiency
FF: Fill factor
IR: Infrared
PL: Photoluminescence
PMT: Photomultiplier tube
SEM: Scanning electron microscope
SLG: Soda-lime glass
